# Cellular receptors for mammalian viruses

**DOI:** 10.1371/journal.ppat.1012021

**Published:** 2024-02-20

**Authors:** Ana Valero-Rello, Carlos Baeza-Delgado, Iván Andreu-Moreno, Rafael Sanjuán

**Affiliations:** Institute for Integrative Systems Biology (I2SysBio), Consejo Superior de Investigaciones Científicas-Universitat de València, Paterna, València, Spain; Boston College, UNITED STATES

## Abstract

The interaction of viral surface components with cellular receptors and other entry factors determines key features of viral infection such as host range, tropism and virulence. Despite intensive research, our understanding of these interactions remains limited. Here, we report a systematic analysis of published work on mammalian virus receptors and attachment factors. We build a dataset twice the size of those available to date and specify the role of each factor in virus entry. We identify cellular proteins that are preferentially used as virus receptors, which tend to be plasma membrane proteins with a high propensity to interact with other proteins. Using machine learning, we assign cell surface proteins a score that predicts their ability to function as virus receptors. Our results also reveal common patterns of receptor usage among viruses and suggest that enveloped viruses tend to use a broader repertoire of alternative receptors than non-enveloped viruses, a feature that might confer them with higher interspecies transmissibility.

## Author summary

Specific interactions between viruses and cellular entry factors are a critical initial step in viral infection. The identification of virus receptors and other key entry factors is important for understanding viral tropism, pathogenesis and cross-species transmissibility, as well as for the design of antiviral drugs. Here, we provide a comprehensive meta-analysis of our current knowledge of virus receptors and attachment factors. We use the newly assembled dataset to reveal general patterns of receptor use across viruses and to derive predictions about the propensity of each cell surface protein to serve as a virus receptor. This work may assist future research on receptor discoveries, and suggests new implications of virus-receptor interactions, including viral emergence.

## Introduction

Mammals can be infected by thousands of viruses belonging to tens of different families. Cellular receptors and other entry factors critically determine infectivity and play a major role in viral cross-species transmission [1–4]. There are numerous examples showing the evolution of key mutations in viral receptor-binding proteins that promote transmissibility. For instance, certain changes in the influenza virus hemagglutinin determine sialic acid preferences and the ability of avian strains to infect humans [5,6]. Similarly, changes in the affinity of the viral spike protein for human ACE2 have been instrumental in the emergence and evolution of SARS-CoV-2 [7,8]. Conversely, several receptor-coding genes have evolved under virus-driven selection, such NPC1 in bats [9] and TFRC in rodents [10], among others. Therefore, the identification of cellular factors involved in viral entry is a cornerstone in our understanding of viral tissue tropism and pathogenesis. The discovery and characterization of virus receptors also facilitate the development of entry inhibitors and allow the targeting of therapeutic viruses to specific cells [11].

However, virus receptor studies can be technically challenging, particularly due to the complex nature of viral entry, which often involves redundant receptors, co-receptors, and accessory receptors, as well as different attachment factors. The use of multiple functional receptors by viruses has been extensively documented, two examples being SARS-related [12,13] and Zika [14] viruses. Strategies for the identification of cellular factors determining viral entry include systematic perturbation methods such as RNAi, CRISPR-Cas, and overexpression of candidate genes, as well as biochemical and biophysical methods used to demonstrate and quantify virus-receptor binding, including protein microarrays, affinity-purification mass spectrometry, biolayer interferometry, and plasmon resonance [15,16]. Virus receptor inference can involve full viruses or use pseudotypes, an approach that focuses precisely on viral entry and allows handling non-culturable viruses [17].

Currently, virus receptors are collected in a few databases, such as ViralZone (viralzone.expasy.org), KEGG (www.genome.jp/kegg/), and VTHunter (db.cngb.org/VThunter). Moreover, previous articles have relied on this information in combination with different search strategies to investigate viral entry factors [18–21]. Overall, these databases and previous works report about 100 cellular receptors for a similar number of viruses. However, they may not provide a comprehensive view of the literature, and their content is probably biased towards human and economically relevant viruses. Here, we aim to provide a more thorough analysis of the actual diversity of known receptors and attachment factors used by mammalian viruses. To achieve this goal, we implemented systematic and semi-automated search strategies that allowed us to double the amount of information extracted from the literature compared to previous work, and to pinpoint the role of each cellular factor in viral entry. Using machine learning, we identify cell surface proteins that are more likely to function as virus receptors, as well as common features of these proteins. We also explore how the repertoire of cellular receptors varies according to viral species, families and other viral features, and show that this repertoire correlates with viral cross-species transmissibility.

## Results

### Dataset of virus receptors

**Fig 1:**
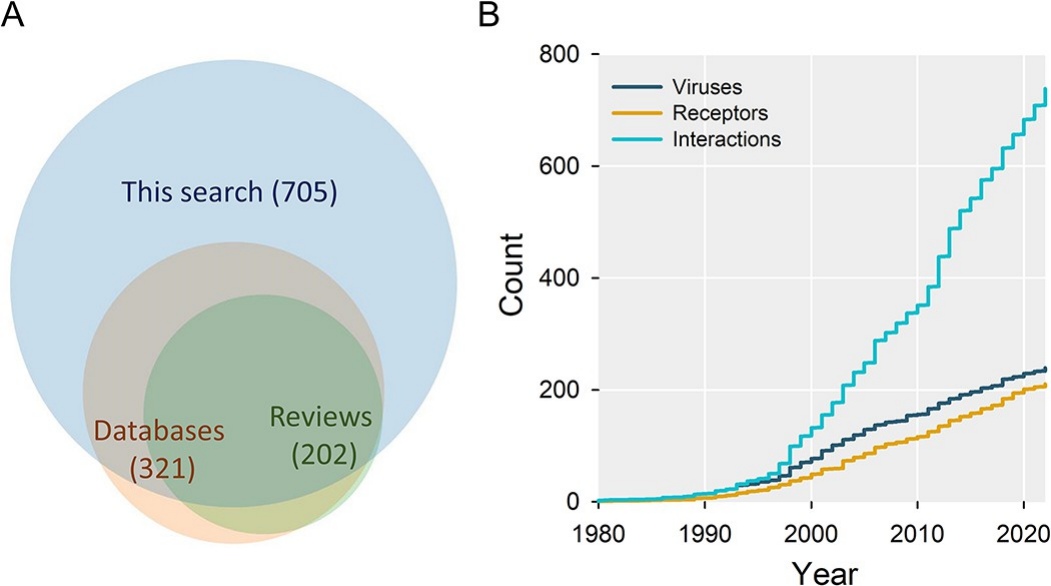
Virus receptor discovery trends. **A.** Venn diagram showing the number of virus-receptor interactions in three databases combined (ViralZone, KEGG, VTHunter), two previous reviews or meta-analyses (see text), and the search performed in this study. Areas are proportional to the number of interactions described, and numbers indicate the total number of pairs from each source. See Tables 1 and S1 for details. **B.** Cumulated numbers of distinct virus-receptor interactions, viruses, and host factors discovered per year.

We defined receptors as host factors promoting viral entry through direct interactions with the surface of viral particles. This includes receptors believed to be both necessary and sufficient for viral entry, but also alternative receptors (sufficient but not necessary for viral entry), co-receptors (necessary but not sufficient), and accessory receptors (promoting entry but not necessary or sufficient). We also included attachment factors, defined as moieties that promote initial virus binding. A PubMed search of mammalian virus names associated with the keywords “receptor”, “entry”, “binding”, or “attach” yielded 67,492 results, which were filtered and analyzed using a combination of automated text mining and manual revision of abstracts or full-length articles when needed (**S1 Fig**). This allowed us to retrieve 705 distinct virus-host interactions involving 233 viral species and 204 cellular factors. Of these, 61.3% were identified by the manual approach, 7.7% by the automated method, and 31.0% by both methods. Compared to three previous databases (ViralZone, KEGG, VTHunter) and two previous meta-analyses [18,20], our search increased by 2.2-fold the number of interactions, while capturing 95.5% of those reported in these other sources (**Fig 1A and Table 1**). After pooling all data, we obtained a final dataset consisting of 210 cellular factors, 239 viral species, and 738 interactions. For each of these, we provide the name of the virus, the name, chemical nature (protein, carbohydrate, lipid) and functional role of the cellular factor (main receptor, alternative receptor, co-receptor, accessory receptor, or attachment factor), the gene symbol for protein-coding genes, the PMID of the original publication, year of discovery, and whether the interaction was reported in previous reviews and databases (**S1 Table**). Analysis of publication years of the original articles showed that virus receptor discovery rates have been approximately constant since 2000, with roughly 7.6 new cellular factors, 7.7 new viruses, and 28.2 new interactions per year, the latter rate showing a slight acceleration in recent years (**Fig 1B**).

**Table 1:** Numbers of distinct host factors, viruses, and virus-host interactions collected in this study as well as previous publications and databases.

	Host factors	Viruses^g^	Interactions^g^
KEGG^a^	75	89	174
ViralZone^b^	76	105	215
VTHunter^c^	87	105	215
Zhang et al.[20]^d^	69	94	176
Wang et al.[18]^d^	81	91	190
Combined^e^	112	140	324
New search	204	233	705
Fold increase^f^	1.8	1.7	2.2
Total	210	239	738

^a^Extracted from genome.jp/kegg

^b^From viralzone.expasy.org/5356

^c^Extracted from db.cngb.org/VThunter

^d^See text for reference.

^e^Combination of all the above sources.

^f^This study compared to the five other sources of information combined.

^g^Five viral species were split into 11 subgroups as the pattern of their receptor usage was markedly different.

Of the 738 virus-host interactions identified, 616 involved 201 receptors constituted by host proteins or protein complexes. Overall, 22.2% corresponded to main receptors, 22.5% to alternative receptors, 10.7% to co-receptors, and 28.0% to accessory receptors (**Table 2**). However, these assigned roles can be uncertain, since they depend on how data were interpreted in the original publications. For instance, a receptor might be misclassified as sufficient for entry if experimental evidence was obtained in cells that expressed an unknown co-receptor. Also, the main receptor could be functionally equivalent to alternative receptors, the only difference being the time of discovery. Additionally, a given cellular protein could play different roles depending on the virus. The remaining 122 interactions (16.5%) corresponded to 9 different moieties linked to unspecified proteins. In most cases, these moieties were sialic acids or other glycans such as heparan sulfate. For some viruses such as influenza virus, the molecular details of the interaction between carbohydrates and viral surface proteins have been characterized extensively, but in most cases such details are unknown. Moreover, carbohydrate moieties typically function as attachment factors, such that viral particles bind to the carbohydrate molecules but may not enter the cell unless these moieties are found in a cell surface protein capable of mediating viral internalization.

**Table 2:** Virus-receptor interactions classified according to the nature and role of the host factors involved, showing differences between enveloped and non-enveloped viruses.

Nature	Role	Total	Enveloped	Non-enveloped
Proteins	Main receptor	164 (22.2%)	127 (21.8%)	37 (23.7%)
	Alternative receptor	166 (22.5%)	146 (25.1%)	20 (12.8%)
	Co-receptor	79 (10.7%)	67 (11.5%)	12 (7.7%)
	Accessory receptor	207 (28.0%)	174 (29.9%)	33 (21.2%)
Moieties	Attachment	122 (16.5%)	68 (11.7%)	54 (34.6%)
Total		738 (100%)	582 (100%)	156 (100%)

### Overview of the patterns of receptor use across viruses

**Fig 2:**
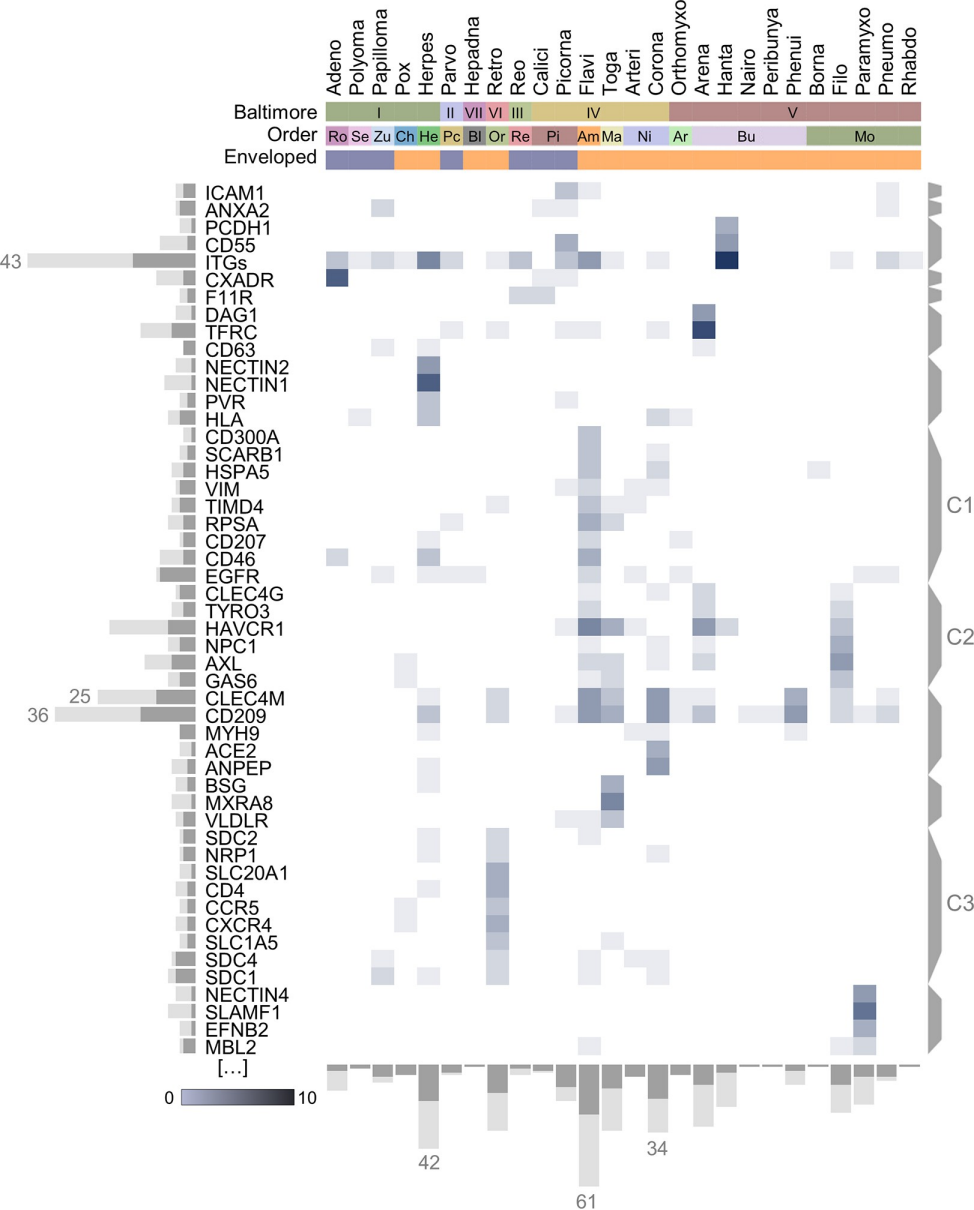
Heat map of the 50 cellular proteins most frequently used as receptors by mammalian viruses. HUGO gene names are shown, except for the HLA, VGGC, and integrin (ITGs) complexes. Shades of grey indicate the number of viruses known to use each protein as a receptor. Bars on the left show the total number of viruses (light grey) and viral families (dark grey) using each protein (top three values indicated). Brackets on the right indicate protein clusters obtained in a hierarchical cluster analysis, using the cosine similarity metric to group proteins according to levels of virus sharing. Three such clusters (C1-C3) are highlighted. In columns, viruses are aggregated by viral families. Baltimore group, taxonomical order, and whether the virus is enveloped are indicated. For each family, grey bars at the bottom show the number of known virus-receptor pairs (light grey; top values indicated), and the number of distinct receptors used (dark grey). Ro: Rowavirales; Se: Sepolyvirales; Zu: Zurhausenvirales; Ch:Chitovirales; He: Herpesvirales; Pc: Piccovirales; Bl: Blubervirales; Or: Ortervirales; Re: Reovirales; Pi: Picornavirales; Am: Amarillovirales; Ma:Martellivirales; Ni: Nidovirales; Ar: Articulavirales;Bu: Bunyavirales; Mo: Mononegavirales.

Among the total 201 individual proteins or protein complexes identified, some were used by many viruses, the most frequent being different integrin subunits, followed by CD209 (DC-SIGN) and CLEC4M (C-type lectins), HAVCR1 (TIM-1), TFRC and AXL, each associated to >10 different viruses belonging to more than five families (**Fig 2**). As previously noted [3,22], this underscores that viral entry frequently exploits cellular functions related to cell-cell adhesion (e.g. integrins), carbohydrate-mediated signalling (lectins), and autophagy (HAVCR1, AXL), and shows that non-proteinaceous components of the virion surface can play a central role in this process. For instance, carbohydrates in viral surface glycoproteins can bind lectins [23–25], and HAVCR1 can interact with the lipid membrane of many enveloped viruses to promote viral endocytosis in a process known as apoptotic mimicry [26,27].

A hierarchical cluster analysis in which entry factors were grouped according to virus sharing suggested some general patterns (**Fig 2**). A large group (C1) was formed by host proteins with heterogeneous functions used predominantly by plus-strand RNA viruses, particularly flaviviruses, coronaviruses, and togaviruses. Another cluster (C2) included AXL, TYRO3, HAVCR1, and NPC1, which are frequent receptors for flaviviruses, togaviruses, arenaviruses, and filoviruses. These viruses are often internalized nonspecifically in cells by apoptotic mimicry and use downstream specific receptors that mediate membrane fusion in the endosome, such as NPC1. Other receptor clusters were associated with a given viral family, such as solute carriers and immune signalling proteins for retroviruses (C3).

### Predictability of virus receptors

**Fig 3:**
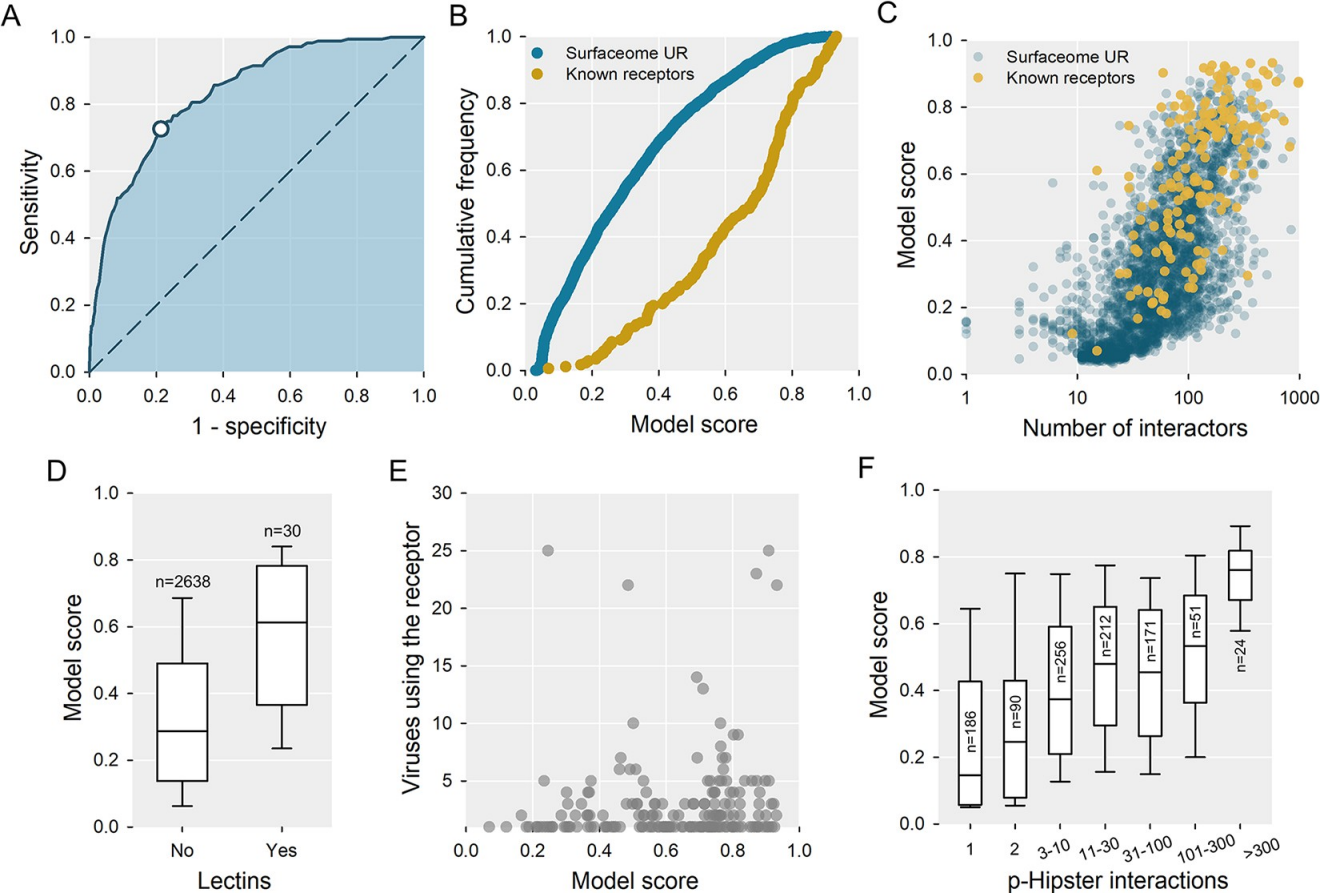
Predictability of virus receptors. **A.** AUC plot of the selected model. The white dot shows the sensitivity and specificity achieved when a threshold score of 0.5 was used to classify cell surface proteins as virus receptors. **B.** Cumulative frequency plots of the scores obtained for 175 known receptors included in the model and 2668 surfaceome proteins not known to be virus receptors (surfaceome UR). **C.** Model scores against the number of protein interactors, measured as the degree parameter in the STRING database. **D.** Model scores for the subset of surfaceome proteins classified as lectins versus non-lectins. This subset was obtained from unilectin.expasy.org/humanLectome, selecting curated lectins only. The number of proteins in each group is indicated. **E.** For receptors known to be used by at least one virus, relationship between the model score and the actual number of viruses using this receptor. **F.** Relationship between the model score and the number of interactions with viruses predicted by p-Hipster (phipster.org). This information was available for 990 surfaceome proteins. For visualization, the number of p-Hipster interactions was categorized into the indicated groups. Group sizes are shown.

We set out to explore features that could predict whether a protein may serve as a virus receptor. For this, we compared 175 known receptors with 2668 plasma membrane proteins located at the cell surface (surfaceome) that have not been previously involved in viral entry. Using a generalized boosted model (GBM), we analyzed a large number of features including functional domains (PFAMs), expression profiles in 54 healthy human tissues, protein size, the number of human protein interactors, post-translational modifications (glycosylation, lipidation, disulfide bonds), sequence distance and synonymous to nonsynonymous evolution rate ratios (dN/dS) between human proteins and their orthologs in four mammal species, and >13,000 Gene Ontology annotation terms, excluding virus-related functions. Our best model showed an AUC of 84.0%, improving the performance of previous models [28] (**Fig 3A**). Of the 12 proteins with a score >0.90, 11 were known receptors and, overall, the distributions of scores assigned to known receptors and the rest of surfaceome proteins were well differentiated (**Fig 3B**). Using a threshold score of 0.5, the model detected 72.6% of the known receptors, but also predicted 570 that have not been so far described (**S2 Table**). These could be false positives, but also undiscovered receptors. The 20 proteins with the highest scores among those not known to be receptors are shown in **Table 3**.

**Table 3:** List of the proteins with the highest GBM scores among surfaceome proteins currently unknown to function as virus receptors.

Gene symbol	Description	GBM score
APP	Amyloid beta precursor protein	0.914
ENG	Endoglin	0.897
CSF3R	Colony Stimulating Factor 3 Receptor	0.891
THBS1	Thrombospon-din 1	0.890
CD44	Hyaluronate Receptor	0.886
CXCR3	C-X-C Motif Chemokine Receptor 3	0.882
CD33	Sialic Acid-Binding Ig-Like Lectin 3	0.881
CD59	CD59 Molecule, Complement Regulatory Protein	0.880
ALCAM	Activated Leukocyte Cell Adhesion Molecule	0.879
CD226	Platelet And T Cell Activation Antigen 1	0.873
RHOB	Ras Homolog Gene Family, Member B	0.870
AOC3	Amine Oxidase, Copper Containing 3 (Vascular Adhesion Protein 1)	0.868
ITGB4	Integrin Subunit Beta 4	0.867
MCAM	Melanoma Cell Adhesion Molecule	0.863
CD22	Sialic Acid-Binding Ig-Like Lectin 2	0.846
ATP1B1	ATPase Na+/K+ Transporting Subunit Beta 1	0.843
HLA-E	Major Histocompatibility Complex, Class I, E	0.843
SELL	Selectin L	0.842
STAB1	Stabilin 1	0.840
MME	Membrane Metalloendopeptidase	0.840

Protein features were ranked according to their relative importance in the model (**S3 Table**), the top single variable being the number of protein interactors (16.0% gain; **Fig 3C**), followed by the GO term “protein binding” (GO.0005515; 8.3% gain). This underscores that the more exposed location of highly-interacting proteins to be accessible to their ligands also makes them more accessible to viruses [20]. Indeed, many proteins that bind or transport other ligands are virus receptors, and it is known that cell adhesion proteins are preferred receptors for viruses [29]. Consistently, the “cell adhesion” GO term (GO.0007155) was the third most important variable in the model (gain 6.6%). Collectively, the gene expression levels in 54 human tissues were a highly relevant feature (46.1% gain), although none of them reached a high value individually (<6.3% gain), meaning that proteins expressed at high levels in at least some tissues are more likely to be receptors. Other features were frequently used for classification (high cover) although they were not among the most decisive features. This included having a high density of disulfide bonds, high glycosylation, containing Immunoglobulin domains (PFAM term PF07686), or participating in cell chemotaxis (**S3 Table**). The role of protein glycosylation was expected considering that many viruses are known to use carbohydrates as attachment factors [25,30]. Conversely, although GO terms associated with lectins did not appear among the most important variables in the model, lectins tended to show higher scores than other proteins (**Fig 3D**). This was also expected because the binding of cellular lectins to carbohydrates present in viral particles can promote viral entry [31]. Finally, despite virus receptors being frequently under positive selection [18], dN/dS ratios and divergence values among mammals did not feature among the top variables of the model (<1% gain).

The quality of the predictions was supported by two additional observations from information not used for training the model. First, the model used binary information about whether or not each protein is a known virus receptor but did not consider how many different viruses have been shown to use each receptor. Nevertheless, we found a weak but significant correlation between the model score of known receptors and the log number of viruses reported to use each receptor (Pearson r = 0.186, P = 0.009; **Fig 3E**). As a second external check of the model, for each surfaceome protein we obtained the number of interactions with viruses predicted in p-Hipster (phipster.org) [32]. The algorithms used for p-Hipster predictions are structured-based and hence do not use the same type of information as our model. Despite this, we found a significant correlation between the log number of predicted interactions in p-Hipster and our model score (Pearson r = 0.370, P < 0.001; **Fig 3F**).

### Variations in the type and number of receptors used by different viruses

Our tentative functional classification of receptors suggested that non-enveloped viruses tend to rely on a single receptor more often than enveloped viruses, which on the contrary are more likely to use alternative receptors, each sufficient for entry (**Table 2** and **S2 Fig**). We also found that non-enveloped viruses are three times more often reported to use carbohydrate moieties associated with undefined proteins than enveloped viruses (34.6% versus 11.7% of all interactions, respectively). Dependence on such moieties is seemingly strongest for caliciviruses and polyomaviruses (60.0% and 80.0% of the total interactions, respectively) and weakest for retroviruses (1.3%).

**Fig 4:**
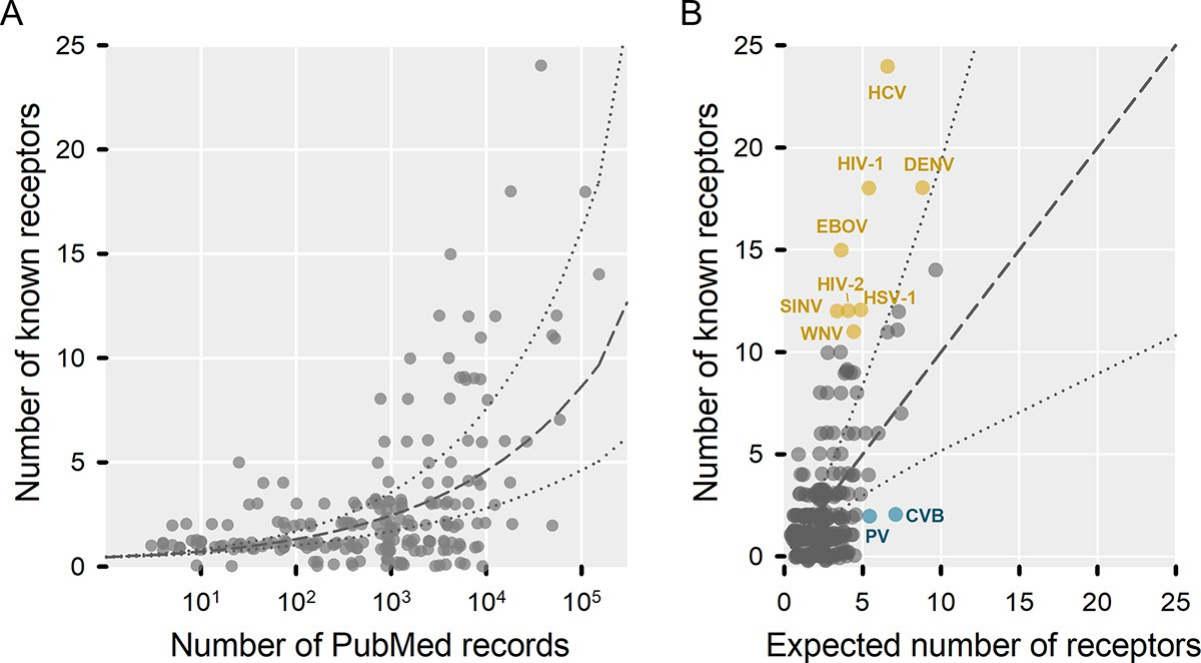
Variation in the number of known receptors across viruses. **A.** Relationship between research effort, measured the number of PubMed records and the number of different proteins used as receptors for each virus. The dashed line shows the expected number of receptors obtained from a GLM (null model), and dotted lines correspond to 95% confidence intervals. **B.** Known receptors versus the expected number under the null model shown in panel A. The dashed line indicates no deviation from the model, and the dotted lines the 95% confidence interval. Viruses shown in yellow exceed the upper limit of the confidence interval and have more than 10 known receptors. Viruses shown in blue fall below the lower limit of the confidence interval, and their expected number of receptors according to the model is higher than five. CVB: Coxsackievirus B (enterovirus B); DENV: dengue virus; EBOV: Zaire ebolavirus; HCV: hepatitis C virus (hepacivirus C); HSV-1: herpes simplex virus 1 (human alphaherpesvirus 1); PV: poliovirus (enterovirus C); SINV: Sindbis virus; WNV: West Nile virus.

We then examined in more detail how many different cellular proteins are used as receptors by each virus. We found ample variation across viral families, with over 50 described for flaviviruses, retroviruses, and herpesviruses, whereas other families such as *Circoviridae*, *Nairoviridae*, *Bornaviridae*, and *Polyomaviridae* showed five or fewer (**Fig 2**). However, these observations can be strongly biased by the research effort dedicated to each virus. To address this, we used the number of publications in PubMed as a proxy of research effort, and estimated the effect of this variable on the known number of proteins used as receptors by each virus, using a generalized linear model (GLM; **Fig 4A**). This allowed us to identify viruses that use more receptors than expected from research effort alone, such as hepatitis C virus, dengue virus, HIV-1 and HIV-2, and Ebola virus (**Fig 4B**). In some cases, such as HIV-2, the excess of known receptors could be explained by knowledge acquired from a related virus, whereas other viruses may be subject to research biases not accounted for here, or might be truly promiscuous in terms of receptor usage. Some highly studied viruses did not exhibit particularly high numbers of host proteins used as receptors, such as influenza viruses, and some enteric viruses. Specifically, according to our search, no cellular proteins have been found to serve as receptors for influenza B or Norwalk viruses despite these being well-studied viruses research and the fact that several receptors have been described for the related influenza A virus and murine norovirus, respectively.

To test for more general patterns, we added to our GLM two more factors: the viral family, and whether or not the virus is enveloped. This showed that, overall, enveloped viruses use a wider variety of host proteins as receptors than non-enveloped viruses, and also more receptors that are sufficient for entry according to the literature (main or alternative receptors; P < 0.001). We inferred from this model that, after controlling for research effort, enveloped viruses use on average 2.4 times more cellular proteins as receptors than non-enveloped viruses. This excess was more marked for phenuiviruses, togaviruses, and filoviruses, whereas polyomaviruses and caliciviruses showed particularly low numbers of such receptors (**S3 Fig**).

### The repertoire of alternative receptors correlates with the viral host range

**Fig 5:**
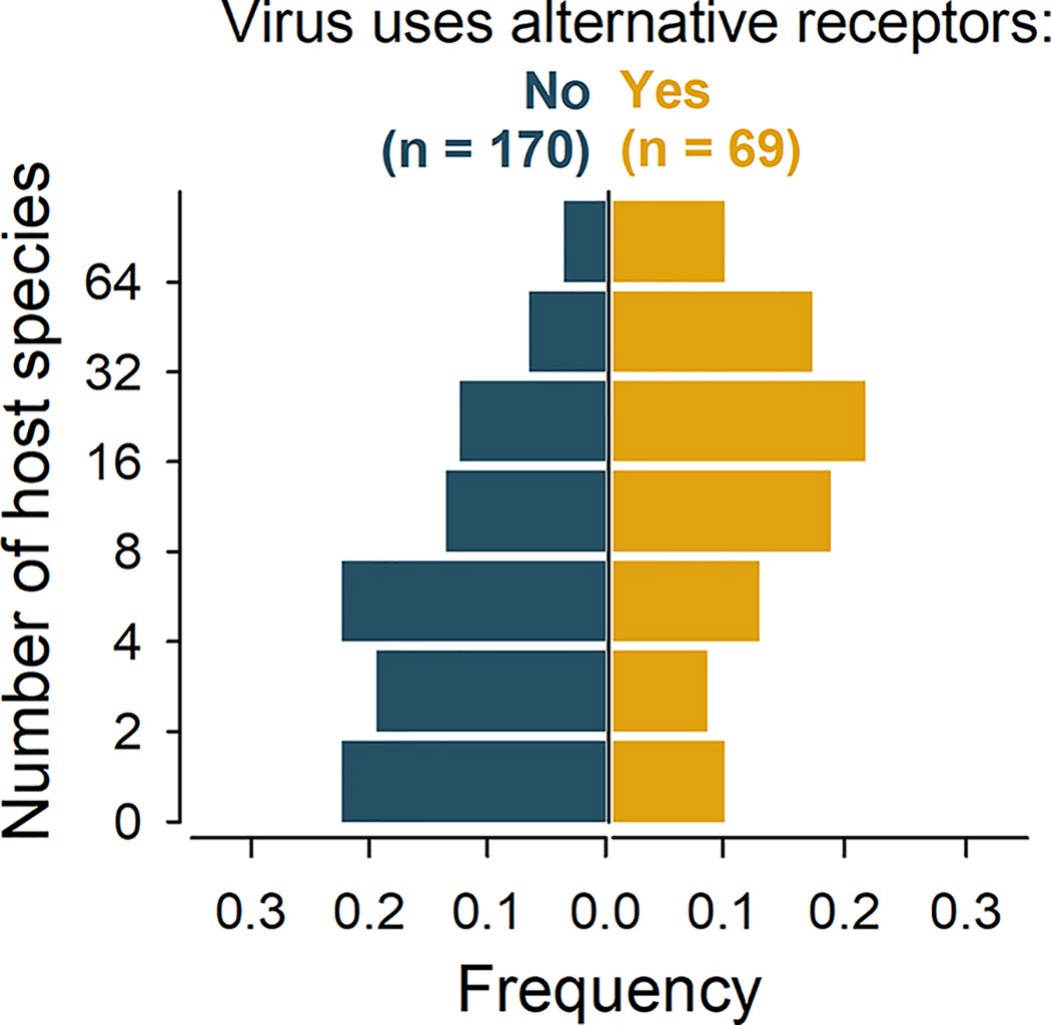
Relationship between viral host range and use of alternative receptors for viral entry. The distribution of the number of host species per virus is shown for viruses for which there is only one known protein receptor functionally sufficient for entry, versus those with alternative receptors. Virus-host associations were obtained from a previous publication (see text).

In a previous publication, we used known virus-host associations to show that enveloped viruses infect on average a larger number of different host species than non-enveloped viruses [33]. Since we have shown here that enveloped viruses also tend to display a broader repertoire of receptors, we reasoned that the ability to use alternative receptors could allow viruses to infect more host species. Interspecies variability in receptor genes can prevent cross-species transmission, but this barrier might be less stringent if a virus can use other receptors that are also sufficient for entry. We found that the average number of host species was over twofold higher for viruses known to use alternative receptors (26.7 ± 4.2) than for viruses using only one receptor (11.6 ± 1.4; **Fig 5**). However, this might be again due to differences in research effort. To address this, we implemented a GLM in which, as above, the number of PubMed records per virus was used to quantify research effort. This confirmed that the use of alternative receptors is associated with the ability to infect a larger number of host species (P < 0.001), with an estimated 1.6-fold increase in the number of host species per virus. Therefore, the broader host range shown by enveloped viruses might be in part attributed to their greater tendency to use alternative receptors, compared to non-enveloped viruses.

## Discussion

By performing a systematic search of virus receptors, we have provided a general overview of the state of the art in this area of research and identified knowledge gaps. Our dataset may assist future research on receptor discovery, antiviral therapeutics, and virus-host interactions. The search was restricted to mammalian viruses due to their relevance, but also due to current limitations in our ability to perform more generalized analyses that would also include other vertebrate, invertebrate, plant, fungal, and prokaryotic viruses. Despite being as exhaustive as possible, our search strategy was mostly based on reviewing article titles, abstracts, and MESH terms, whereas full-length documents were analyzed only in cases of uncertainty. The automated text mining approach allowed us to ensure objectivity and to expand our search capacities, but was also based on abstracts and required that the virus and host gene terms be in the same sentence, limiting its power. Further optimizations of this automated pipeline could include full text, but this would strongly increase computing demands. In addition, we provide information about the role of each cellular protein in viral entry, which can vary from strictly necessary and sufficient to an accessory role. However, this classification is challenging, and in several instances, a given protein was classified as an alternative receptor by some authors and as an accessory receptor by others. We attempted to solve these incongruences by using clear definitions and checking raw results in the articles when necessary, but current knowledge gaps inherently limit our ability to robustly assign a functional role to each receptor. Finally, we classified all moieties such as sialic acids and other glycans as attachment factors, since their role in viral entry is probably dependent on the protein to which these moieties are linked. For instance, it has been shown that influenza virus infection depends on attachment to sialic acids, but that entry is triggered by host proteins such as EFGR [34], CACNAC1 [35], or CEACAM6 [36].

High throughput methods such as CRISPR-Cas knockout libraries are often used to select candidate receptor genes, but these require subsequent experimental testing, which can be a complex task. Our machine-learning model could help researchers prioritize candidates at this stage of receptor discovery. According to this model, approximately 700 proteins located at the plasma membrane may function as virus receptors for mammalian viruses, of which most remain unreported. The latter could be real but undiscovered receptors, host proteins that are currently not used by any virus but could potentially serve as receptors, or incorrect predictions of the model. In agreement with previous reports, plasma membrane proteins with functions related to ligand-binding, cell-adhesion, and a high interaction degree are more prone to be used as receptors by mammalian viruses [20,29]. Regardless of the accuracy of these predictions, our data show that the yearly discovery rate of virus receptors has not reached a plateau, indicating that there is still room for a substantial expansion of the virus receptorome. It has been estimated that there are approximately 40,000 viruses in nature capable of infecting mammals [37] but, for several viral families, only a few receptors are known. The VirHostNet database, which reports experimentally validated virus-host interactions, lists >10,000 human proteins that physically interact with at least one viral protein [38], and the p-Hipster database predicted approximately 282,000 such protein-protein interactions [32]. We have shown that top-scoring proteins in our model tend to have large numbers of predicted interactions in p-Hipster. Examples include THBS1 (thrombospondin 1) with the norovirus major capsid protein, APP (amyloid beta precursor protein) with influenza A virus hemagglutinin and the envelope protein of several flaviviruses, and NTRK1 with betaherpesvirus membrane glycoproteins.

Overall, we have found that enveloped viruses display a broader repertoire of known receptors than non-enveloped viruses. Some properties of viral envelopes could explain such differences. Envelope proteins might be structurally less constrained than capsid proteins, which form highly rigid and complex structures with potentially lower ability to accommodate different types of interactions with cellular proteins. Relatedly, this might also allow envelope proteins to accumulate increased genetic diversity, facilitating their evolution toward new receptor usages. We also found that viruses that use alternative receptors tend to show broader host ranges. These findings are consistent with our previous observation that enveloped viruses exhibit a greater propensity to cross-species transmission and zoonosis than non-enveloped viruses [33]. In part, the larger number of virus-receptor interactions found in enveloped viruses could in principle be a result of differences in research effort, but this confounder was accounted for in our analyses.

In conclusion, this work may promote future research in the field by providing a comprehensive dataset of known virus-host interactions involved in viral entry, which could be probed in related viruses, and by suggesting candidate cell surface proteins for virus receptor discovery. It also reveals previously unrecognized differences in the usage patterns of cellular entry factors among viruses, viral families, and major viral groups.

## Methods

### Manual search strategy

A list of 6034 viruses known to infect mammals, available from previous work [33], was used as a query. NCBI Entrez was interrogated for publications meeting the following criteria (**S1 Fig**): (i) to contain the name of the query virus, or any of their aliases obtained from NCBI Taxonomy, coded as “organism”; (ii) to contain the keywords “receptor”, “entry”, “binding”, or “attach” in the title, abstract or MESH terms; (iii) publication date later than 1999; (iv) not including the terms “clinical”, “trial”, “therapy”, “therapeutics”, “cohort”, “biomarker”, “RNA-binding”, or “DNA-binding”. The resulting dataset comprised 67,492 articles encompassing 503 viral species. For 423 viruses that had less than 100 articles each, we performed a manual review (6257 total articles), whereas for the 80 viruses with more than 100 articles (61,235 total articles) we carried out a search strategy based on analysis of selected reviews.

### Automated text mining strategy

Our pipeline was inspired by pubmedKB [39]. The above list of 67,492 abstracts was preprocessed by replacing acronyms with their original references in the text, and abstracts were divided into sentences. All resulting sentences were analyzed using the entity recognition and normalization tool BERN2 [40]. Based on the entities labelled by BERN2 as species or diseases, a search was performed in the NCBI Taxonomy database [41] to identify those corresponding to viruses and normalize their names. Sentences containing both virus and gene entities were then analyzed using the two-relations extraction models OpenIE (stanfordnlp.github.io/CoreNLP) and Spacy (spacy.io). The relationships obtained from both models were manually curated to determine which ones correctly identify actual cellular receptors used for viral entry. The complete pipeline is available at github.com/cbaezadelgado/Viral_receptors.

### Additional virus receptor datasets

Our database was completed by extracting receptors from ViralZone (viralzone.expasy.org), KEGG (www.genome.jp/kegg/annotation/br03220.html), QuickGO (ebi.ac.uk/QuickGO/annotations), and three previous meta-analyses [18–20].

### Database curation

The database was manually curated as follows. First, virus taxonomy was normalized according to NCBI Taxonomy, and viruses were grouped into species representatives, which agglutinates all receptors used by its members. For groups of viruses using very different receptor profiles within a species, the groups were kept separated. Endogenous viruses were removed. Second, ambiguous protein names, mammalian receptors without human orthologs, and moieties that are already known to be part of glycoprotein receptors were also removed. Third, only host proteins located at the cell surface were included, with the exception of a few intracellular receptors such as NPC1. The final list of host factors contained 214 protein-coding genes. Of these, 17 encoded integrin subunits, which were pooled into a single receptor class since integrins need to be combined to form functional complexes. Three other complexes with unspecified subunits were included (laminins, HLA, and VGCC), resulting in 201 proteins or protein complexes. In addition, the database included 9 carbohydrate or lipid moieties, thus making 210 total host factors. Virus metadata included taxonomy according to NCBI or ICTV and host data from a curated dataset originally extracted from the VIRION database [33,42].

### Metadata used in the gradient boosting model

The goal of the GBM was to identify plasma membrane-associated proteins located at the cell surface (surfaceome) that are more likely to be used as virus receptors. To define the list of candidate proteins, we first selected human plasma membrane proteins using Gene Ontology (GO) annotation terms GO.0005886 and GO.0009897, which yielded 5112 proteins. We then discarded proteins with the annotation term GO.0009898 (cytoplasmic side of plasma membrane). We also filtered out all pseudogenes listed in GeneCards. Next, among the remaining proteins, we selected only those included in at least one of the following curated databases: (i) the mass spectrometric-derived cell surface protein atlas (wlab.ethz.ch/cspa), the in silico human surfaceome (wlab.ethz.ch/surfaceome), or the cancer surfaceome atlas (fcgportal.org/TCSA). For the latter database, we selected only proteins with a GESP score >4, as recommended by the authors [43]. The curated dataset contained 2843 high-confidence surfaceome proteins, including 175 of the 214 individual proteins known to be virus receptors. For each, we obtained mRNA expression levels in 54 healthy human tissues obtained from the Human Protein Atlas (proteinatlas.org;rna_tissue_consensus.tsv.zip), >13,000 GO terms from Geneontology.org (excluding virus entry-related terms), post-translational modifications (lipidation, glycosylation, disulfide bonds), length, protein families (PFAM) from UniProt (uniprot.org), and the number of human protein interactors in the STRING database (string-db.org). In addition, we calculated the normalized amino-acidic distance between 444 human proteins and their orthologs in four mammal species (*Bos taurus*, *Canis lupus familiaris*, *Mus musculus*, and *Myotis lucifugus*) using the R package ape [44], as well as site (MEME model) [45], and branch-site (aBSREL model) [46] dN/dS ratios of non-synonymous to synonymous evolution rates using Hyphy [47].

### Implementation of the gradient boosting model

An XGBoost classifier was run using the xgboost R package [48]. To address class imbalance, we weighted the positive class (i.e. known virus receptors) by the ratio of the number of negative to positive instances. Then, ten-fold stratified cross-validation was performed to estimate the model predictive ability using a maximum of 10,000 boosting iterations with 50 rounds as an early stopping criterion (xgb.cv function). The area under the ROC curve (AUC) was used as the preferred evaluation metric to determine optimal model complexity. A suitable combination of model hyperparameters was found by Bayesian optimization using the R package ParBayesianOptimization (CRAN.R-project.org/package=ParBayesianOptimization). We optimized the maximum depth of boosted trees (max_depth), the fraction of training samples used to construct each tree (subsample), the fraction of predictors used to construct each tree (colsample_bytree), the learning rate (eta), the L1 regularization term (alpha), the L2 regularization term (lambda) and the lagrangian control for tree split (gamma). Because instances labeled as negatives in our dataset might represent undiscovered virus receptors, false positive predictions may not be so. Therefore, higher recall was preferable to higher precision in selecting the optimal model. To account for this in our hyperparameter optimization, we maximized the combination (or average) of precision and recall. We initialized the Bayesian optimization with 100 random parameter combinations and sampled another 10 parameter sets during each refinement epoch, for a total of 50 epochs. The 50 top-ranked models were run again 100 times to take into account the randomness in model training and data subsets generated for cross-validation. Finally, the model with a significantly larger combined precision and recall was selected, which was determined by performing one-tailed one-sample t-test. Finally, we used the average number of boosting iterations to train a final model with full data (xgboost function), which was then used to analyze the relative importance of the predictor variables (xgb.importance function). The complete pipeline and gene database used for the predictive model are available at github.com/cbaezadelgado/Viral_receptors.

### Generalized linear models

GLMs were used to test for differences in the number of known receptors among viruses. The data were assumed to follow a Tweedie distribution, and a log link function was used. The log number of PubMed records available for each virus was used to control for research effort. To test for differences across viral families, the viral family was added to the GLM, and data corresponding to families with less than 5 viral species were removed. The viral family factor was nested within a binary variable indicating whether the virus is enveloped. A GLM with underlying Tweedie distribution and log link function was also used to test for differences in the number of host species known for each virus. This analysis also controlled for research effort using the log number of PubMed records and included a binary variable indicating whether the virus is known to use multiple receptors each sufficient for viral entry (main and alternative receptors). As an alternative metric of research effort, we used the log number of sequences of each virus deposited in Genbank. All the factors shown to be significant using PubMed records were also significant using Genbank sequences.

## Supporting information

S1 Fig Workflow for the manual and automatic text-mining searches. A starting list of 6034 mammal viruses was used to obtain, which were then reviewed using both manual and PubmedKB-based automated strategies. The resulting virus-receptor pairs were combined with known databases and manually curated (see text for full description). M, manual strategy; PKB, PubmedKB strategy.
(TIF)

S2 Fig Roles of known receptors according to viral family. Only families with at least 10 known virus-host interactions are represented. Families of enveloped and non-enveloped viruses are shown, and within each group, families are sorted by the fraction of known receptors that are sufficient for viral entry (main plus alternative receptors).
(TIF)

S3 Fig Research effort versus the known number of host proteins used as receptors for different viral families. Data points correspond to individual viruses. Those corresponding to the indicated family are shown in color (blue for non-enveloped viruses; yellow for enveloped viruses), and grey points correspond to all other viruses. The colored and grey dashed lines show the GLM prediction obtained specifically for the family and all viruses, respectively. Only families with at least 5 viral species in the dataset were considered.
(TIF)

S1 Table Database of virus receptors generated in this study. The following information is provided: the viral species, number of PubMed records and Genbank sequences available for each virus, viral family, presence of an envelope, number of host species, receptor symbol, receptor nature, functional role of the receptor, corresponding gene symbol, original publication PMID, year of discovery, and whether the virus-receptor interaction was reported in previous reviews and databases.
(XLSX)

S2 Table Scores obtained from the GBM. The gene symbol, assigned score (probability of being a receptor), and whether the corresponding protein is a known receptor are indicated.
(XLSX)

S3 Table Relevant features identified by the GBM. Gain represents the relative contribution of each variable to the model prediction. Cover indicates the relative number of observations that are related to a given variable.
(XLSX)
