# Supplementary figures and images for: Cellular receptors for mammalian viruses
Source: PLoS Pathog. 2024 Feb 20;20(2):e1012021. doi: 10.1371/journal.ppat.1012021 (PMC10906839; doi:10.1371/journal.ppat.1012021)

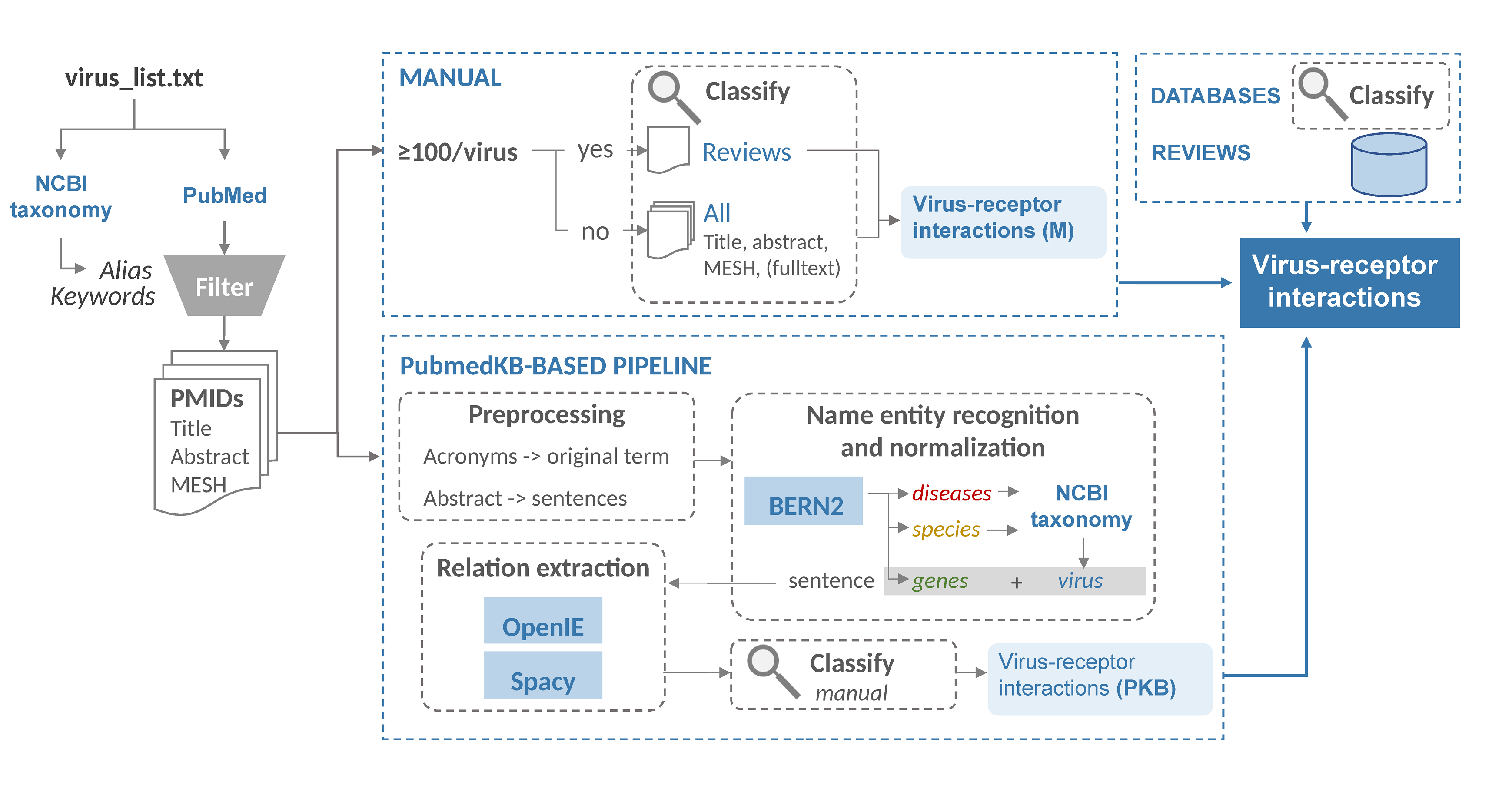

Supplement: S1 Fig — A starting list of 6034 mammal viruses was used to obtain, which were then reviewed using both manual and PubmedKB-based automated strategies. The resulting virus-receptor pairs were combined with known databases and manually curated (see text for full description). M, manual strategy; PKB, PubmedKB strategy. (TIF) [file ppat.1012021.s001.tif]

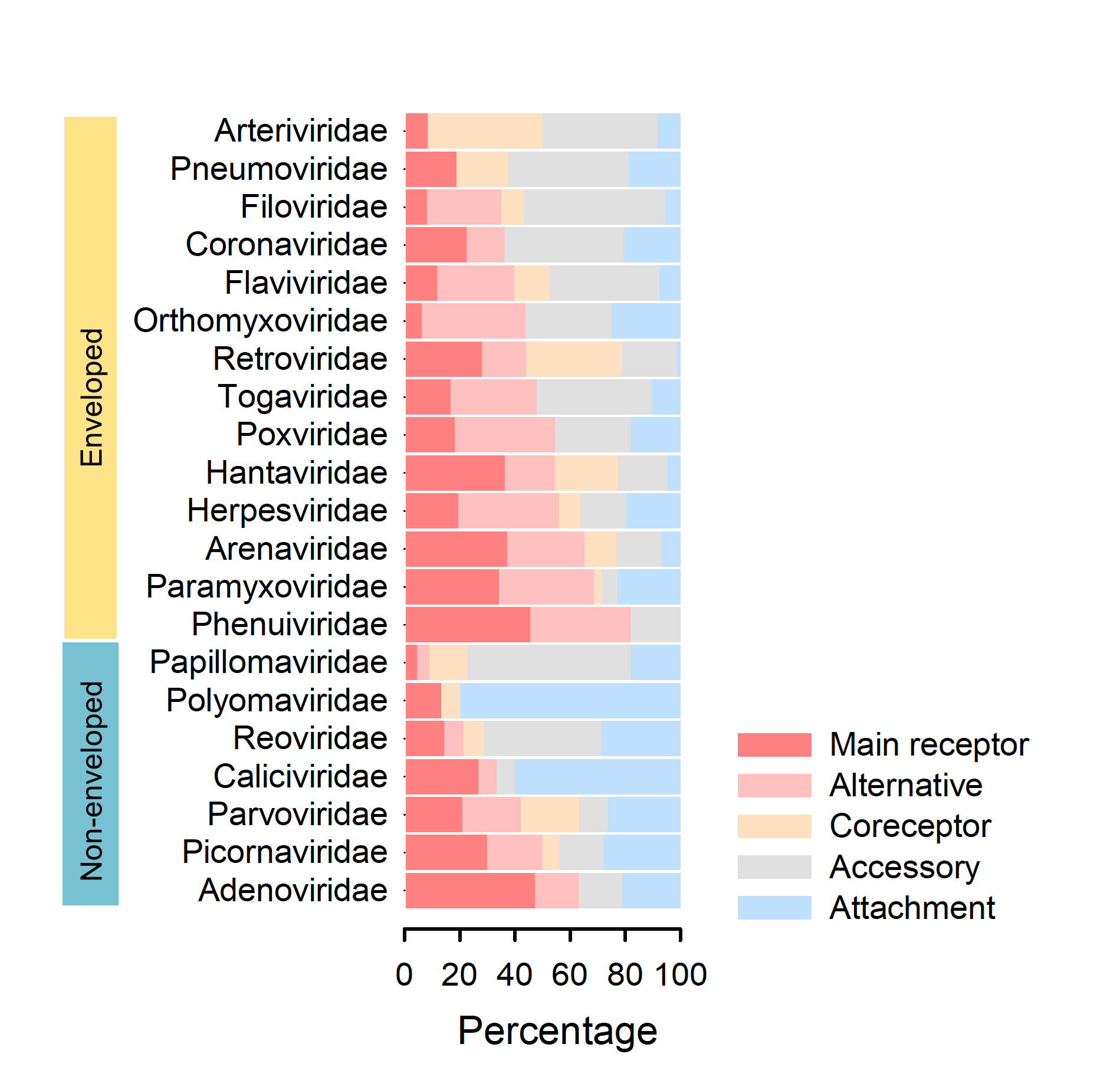

Supplement: S2 Fig — Only families with at least 10 known virus-host interactions are represented. Families of enveloped and non-enveloped viruses are shown, and within each group, families are sorted by the fraction of known receptors that are sufficient for viral entry (main plus alternative receptors). (TIF) [file ppat.1012021.s002.tif]

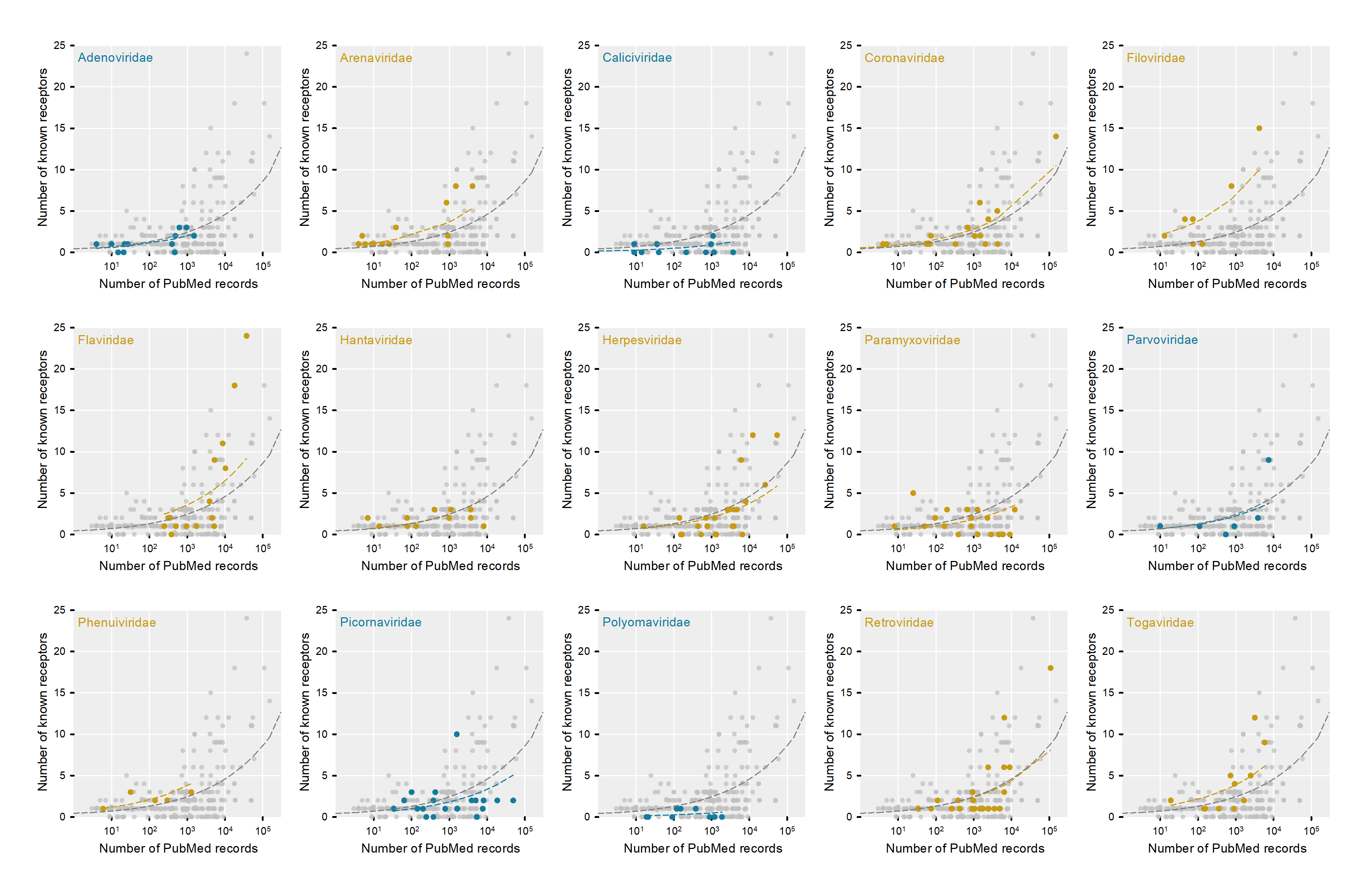

Supplement: S3 Fig — Data points correspond to individual viruses. Those corresponding to the indicated family are shown in color (blue for non-enveloped viruses; yellow for enveloped viruses), and grey points correspond to all other viruses. The colored and grey dashed lines show the GLM prediction obtained specifically for the family and all viruses, respectively. Only families with at least 5 viral species in the dataset were considered. (TIF) [file ppat.1012021.s003.tif]
